# Characteristics of Internal Medicine Physicians Disciplined by Professional Colleges in Canada

**DOI:** 10.1097/MD.0000000000000937

**Published:** 2015-07-02

**Authors:** Jessica J. Liu, Asim Q. Alam, Hanna R. Goldberg, John Justin Matelski, Chaim M. Bell

**Affiliations:** From the Department of Medicine, University Health Network (JJL); Department of Anesthesia, Sunnybrook Health Sciences Centre, Toronto, Ontario, Canada (AQA); Institute of Human Nutrition, Columbia University College of Physicians and Surgeons, New York, New York (HRG); Department of Biostatistics, Dalla Lana School of Public Health (JJM); Department of Medicine (CMB); Institute for Health Policy Management and Evaluation, University of Toronto (CMB); and Department of Medicine, Mount Sinai Hospital, Toronto, Ontario, Canada (CMB).

## Abstract

Physician misconduct is of serious concern to patient safety and quality of care. Currently, there are limited data on disciplinary proceedings involving internal medicine (IM) physicians.

The aim of this study was to investigate the number and nature of disciplinary cases among IM physicians compared with those of other disciplined physicians.

Our retrospective study reviewed information from all provincial Colleges of Physicians and Surgeons (CPS) and compiled a database of all disciplined physicians from 2000 to 2013 in Canada. Disciplinary rate differences (RDs) were calculated for IM physicians and compared with other physicians.

From 2000 to 2013, overall disciplinary rates were low (9.6 cases per 10,000 physician years). There were 899 disciplinary cases, 49 of which involved 45 different IM physicians. IM physicians comprised 10.8% of all disciplined physicians and were disciplined at a lower rate than non-IM physicians, incurring 5.18 fewer cases per 10,000 physician years than other physicians (95% confidence interval [CI] 3.62–6.73; *P* < 0.001). They were significantly less likely to be disciplined for: unprofessional conduct (RD 1.16; CI 0.45–1.87; *P* = 0.001); unlicensed activity (RD 0.78; CI 0.37–1.19; *P* < 0.001); standard of care issues (RD 1.37; CI 0.49–2.26; *P* = 0.002); sexual misconduct (RD 1.65; CI 0.90–2.40; *P* < 0.001); miscellaneous (RD 0.80; CI 0.11–1.50; *P* = 0.020); mental illness (RD 0.06; CI 0.01–0.12; *P* = 0.025); inappropriate prescribing (RD 0.74; CI 0.15–1.33; *P* = 0.010); and criminal conviction (RD 0.33; CI 0.00–0.65; *P* = 0.048). No significant differences were found with respect to unclear violations, fraudulent behavior/prevarication, or offenses involving drugs/alcohol (all RDs less than 0.32). IM physicians were also less likely to incur the following penalties: voluntary license surrender (RD 0.53; CI 0.37–0.69; *P* < 0.001); suspension (RD 2.39; CI 1.26–3.51; *P* < 0.001); retraining/assessment (RD 1.58; CI 0.77–2.39; *P* < 0.001); restriction (RD 1.60; CI 0.74–2.46; *P* < 0.001); other (RD 0.52; CI 0.07–0.97; *P* = 0.030); formal reprimand (RD 2.78; CI 1.77–3.79; *P* < 0.001); or fine (RD 3.28; CI 1.89–4.67; *P* < 0.001). No significant differences were found with respect to revocation or mandated counseling/rehabilitation (all RDs less than 0.46).

Generally, disciplinary rates among physicians were low. Compared with other physicians, IM physicians have significantly lower disciplinary rates overall and are less likely to incur the majority of disciplinary offenses and penalties.

## INTRODUCTION

Physician misconduct is of serious concern to the medical profession and to the public. The overwhelming majority of physicians practice medicine without disciplinary action, but negligence, sexual misconduct, fraud, and failure to maintain accepted standards of care have occurred frequently enough to raise concern. Internationally, overall estimated rates of disciplinary action against licensed physicians range from 0.3 to 2.8%.^1–3^ Patient quality of care rests on the standards of practice upheld by physicians and their governing bodies. Although literature on physician misconduct suggests that only a minority of physicians commit these offenses, any indiscretion that has the potential to jeopardize patient safety and quality of care is of utmost importance and worthy of investigation.^4–7^

Medicine is typically a self-governing profession in most Western nations.^1,4,8,9^ In countries such as the U.S., Canada, Australia, New Zealand, and the United Kingdom, disciplinary proceedings against physicians are imposed by physician-governed regulatory bodies at a state or province level. In the U.S., this responsibility falls under the jurisdiction of state medical licensing boards. In Canada, provincial physician regulatory bodies known as Colleges of Physicians and Surgeons are responsible for governing standards of professional practice. These regulatory bodies investigate complaints of misconduct, set fines, revoke and suspend medical licenses, and enforce remediation or restricted work practices if required. Rulings of misconduct and disciplinary action are made public in print or online publications. Although disciplinary proceedings by physician regulatory colleges are not synonymous with misconduct itself, they are considered a surrogate for physician misconduct and unprofessionalism.^4–7^ Moreover, disciplinary proceedings are likely generalizable between countries as compared to other avenues of inquiry, such as criminal law.

In North America, internal medicine (IM) physicians comprise a significant proportion of the physician workforce. Recent data report that there were almost 200,000 practicing IM physicians in the U.S., and nearly 10,000 in Canada.^10–12^ IM physicians are the second-largest group of physicians in the country (second only to family physicians) and represent approximately 10% of the total physician workforce.^12^ Our study examined disciplinary cases among IM physicians and compared them with non-IM disciplined physicians. We hypothesized that rates of misconduct would be lower for IM physicians compared with the rest of the physician population.

## METHODS

Our retrospective study identified all Canadian physicians who were disciplined between January 1, 2000 and December 31, 2013 by reviewing all publicly available online publications on disciplined physicians published by each provincial CPS for this time period. Demographic information for every disciplined physician was collected, including sex, type of practice license (independent practice versus educational license), medical school of graduation (ie, Canadian/U.S.-trained versus international medical graduate), and medical specialty, specifically noting whether a physician was an internal medicine (IM) or non-IM physician. IM physicians were defined according to the Royal College of Physicians and Surgeons of Canada accreditation status in internal medicine. If IM physicians chose to continue onto further subspecialization (such as cardiology), this was also recorded. In addition, the number of years from medical school graduation to disciplinary action was calculated for each case.

Offenses and penalties incurred by IM physicians were collected and compared with other specialties. When information was not available by reviewing the online disciplinary publications, we referred to the Canadian Medical Directory for that year, and attempts were made via email and regular post to obtain supplementary information from their respective regulatory bodies.^13^ Before 2007, online disciplinary data were not available for New Brunswick, Prince Edward Island, Newfoundland and Labrador, the Yukon and the Northwest Territories. In addition, before 2002, disciplinary data were not available for Alberta. The methodology was similar to those used in previous studies on Canadian physicians.^5–7^

### Disciplinary Findings and Penalties

Disciplinary findings were categorized into 1 of 11 categories in keeping previous studies.^1–2,5–7^ Categories included the following: inappropriate prescribing; conviction of a crime; fraudulent behavior or prevarication; misconduct secondary to mental illness; self-use of drugs/alcohol; sexual misconduct; practice below accepted standard of care; unprofessional conduct; unlicensed activity; miscellaneous findings (including improper maintenance of medical records, breaches of confidentiality, or improper disclosure to patients); and unclear findings.

The subsequent penalties incurred were also reviewed and categorized into 1 of 9 categories: license revocation; voluntary license surrender; suspension; license restriction; mandated retraining/education/assessment; mandated participation in psychological counseling or addiction rehabilitation program; formal reprimand; fine/cost repayment; and other penalty.

### Determination of Physician Years for IM and Non-IM Physicians

Disciplined IM physicians were identified from the database. The number of cases was determined by the total number of physicians disciplined since the development of accessible online regulatory body publications. The number of IM physicians and non-IM physicians were obtained from the Canadian Institute of Health Information and Canadian M.D. Post-Education Registry (CAPER).^11–12^

### Statistics

Rate differences (RDs) between IM physicians and non-IM physicians were computed for the entire study period, as well as each year. In addition, RDs for specific offense types and penalties were calculated for the study period. All reported RDs were standardized to events per 10,000 physician years for interpretability. Along with RDs, 95% confidence intervals and *P*-values (representing evidence against the null hypothesis that the true rate difference is 0) are also provided. The standard threshold of alpha equal to 0.05 was used to assess statistical significance. Statistical analyses were performed using R version 3.0.2 via the *ratedifference* function in the *fmsb* package (http://cran.r-project.org/web/packages/fmsb/fmsb.pdf).

## RESULTS

From 2000 to 2013, there were 899 disciplinary cases involving 780 different physicians. There were 49 cases committed by 45 IM physicians, 827 cases committed by 712 non-IM physicians, and 24 cases committed by unnamed physicians (Table 1). On average, IM physicians accounted for 10.8% of all disciplined physicians for each year of our study period. From 2000 to 2013, in the IM group, there were 4 physicians who were each disciplined twice on separate occasions. Of the non-IM physician group, 712 different physicians were involved in 827 disciplinary cases, accounting for 91.9% of all disciplined physicians, and 87 non-IM physicians who underwent multiple disciplinary proceedings. Approximately 3.1% of all disciplined physicians were not publicly named (24/780), and accounted for 2.7% (24/899) of all discipline cases in the study period, although in 1 instance, the disciplined physician's specialty was known to be non-IM and was therefore considered to be non-IM when calculating frequencies (Table 1).

**TABLE 1:**
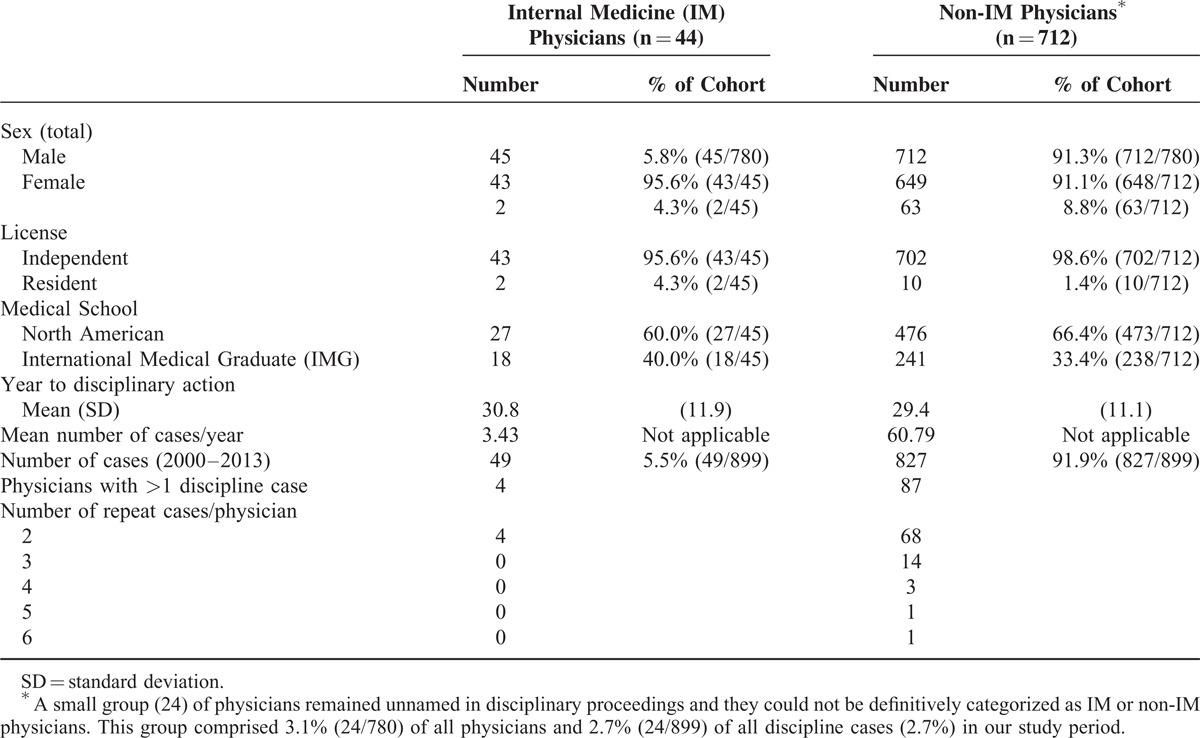
Baseline Characteristics of Internal Medicine (IM) and Non-IM Disciplined Physicians (2000–2013)

The vast majority of disciplined IM physicians were male and independent practitioners (rather than postgraduate trainees) (43/45 or 95.6%). Forty per cent of disciplined IM physicians (18/45 or 40%) were international medical graduates (IMGs; ie, graduated from a non-Canadian/U.S. medical school). Similarly, the vast majority of non-IM physicians were independent practitioners (702/712 or 98.7%), male (712/780 or 91.3%), and approximately one third were IMGs (241/712 or 33.4%). On average, disciplinary action for IM physicians occurred 30.8 years (standard deviation [SD] 11.9) after graduation from medical school, which was similar to non-IM physicians (29.4 years, SD 11.1). General IM was the most frequently disciplined IM subspecialty (14/45 or 31.1%), followed by cardiology (10/44 or 22.2%) (results not shown). Subspecialties such as rheumatology, endocrinology, respirology, gastroenterology, and hematology and medical oncology were also represented but with far less frequency (results not shown). Numerical totals of specific offenses and penalties for IM physicians and non-IM physicians are outlined in Table 1.

**FIGURE 1:**
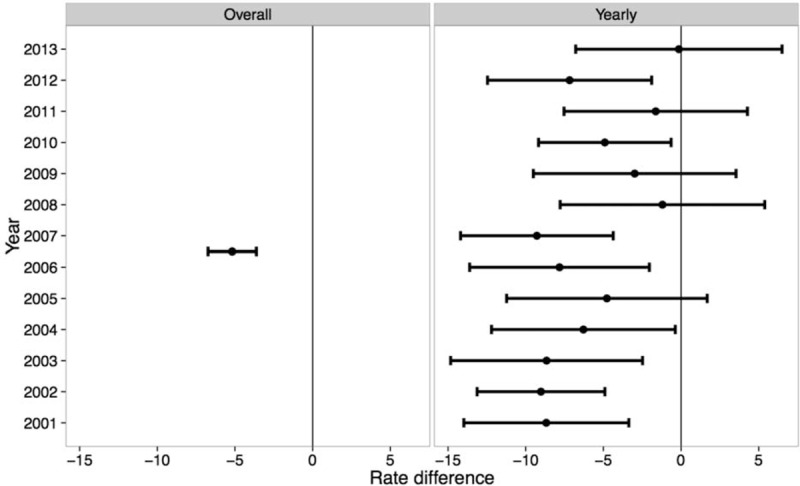
Rate differences (RDs) for all disciplinary cases, by year and cumulative mean, for all internal medicine (IM) physicians, as compared with non-IM physicians^∗^ (2000–2013). ^∗^Error bars denote 95% confidence intervals.

Overall disciplinary rates were low for all physicians. For the study period, the disciplinary rate for all physicians (all specialties) was 9.6 cases per 10,000 physician years. IM and non-IM physicians were disciplined at a rate of 4.98 and 10.15 cases per 10,000 years, respectively (Table 2). For each year in the study period, disciplinary rates were calculated for both groups (Table 2, Figure 1). For 8/14 years, IM physicians were less likely to undergo all disciplinary proceedings as compared with non-IM physicians (no RD lower than 4.9 events per 10,000 physician years). No significant differences were detected between groups for the remaining 6/14 years; however, a rate difference over the cumulative 14-year study period demonstrated that IM physicians incur 5.18 fewer disciplinary cases than non IM physicians per 10,000 physician years (95% CI 3.62–6.73; *P* < 0.001) (Table 2, Figure 1).

**TABLE 2:**
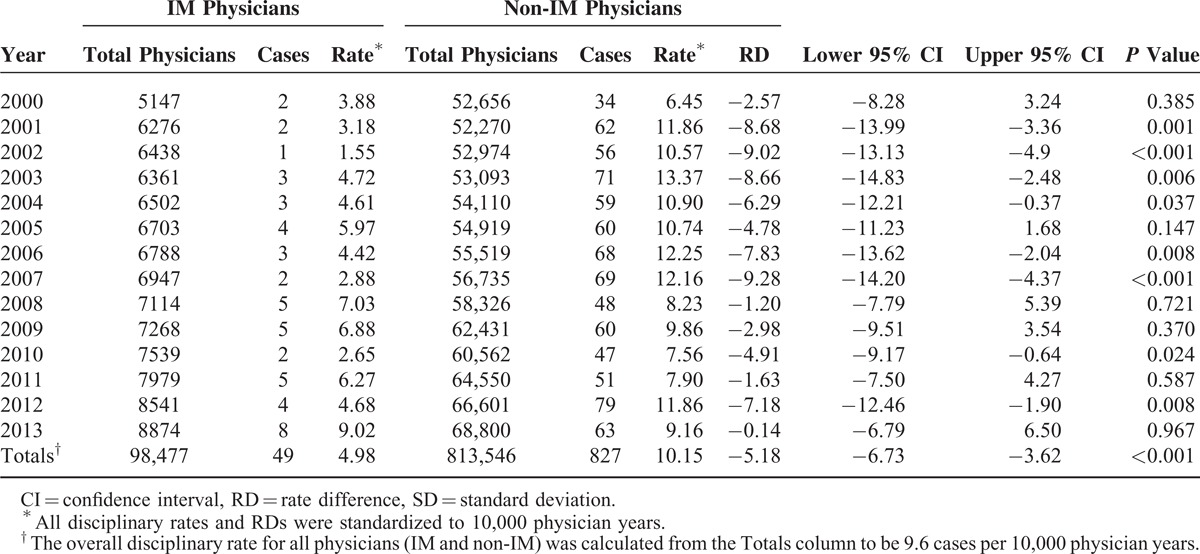
Disciplinary Rates^∗^ and Rate Differences (RDs) for Internal Medicine (IM) Versus Non-IM Physicians, by Year (2000–2013)

**FIGURE 2:**
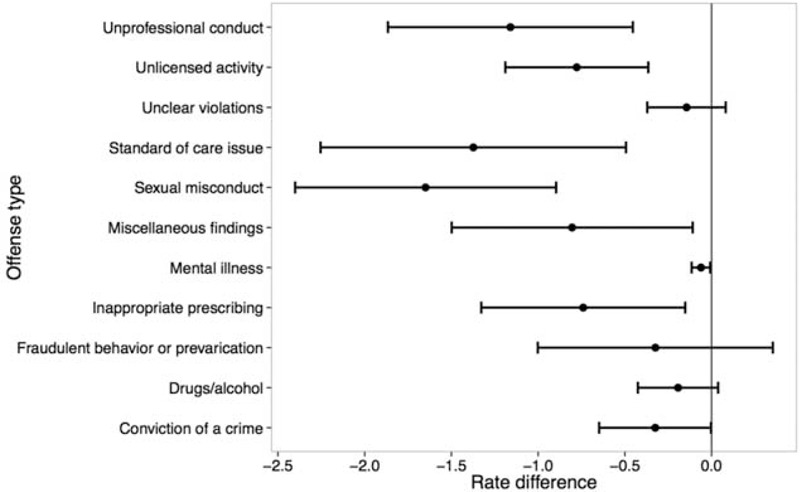
Rate differences (RDs) by disciplinary offense type for internal medicine (IM) physicians, as compared with non-IM physicians^∗^ (2000–2013). ^∗^Error bars denote 95% confidence intervals.

Rates and rate differences of specific offenses and penalty types were calculated for IM physicians and non-IM physicians. IM physicians were significantly less likely than other physicians to be disciplined for the following offenses: unprofessional conduct (1.16 fewer IM cases than non-IM cases per 10,000 physician years; 95% CI 0.45–1.87; *P* = 0.001); unlicensed activity (0.78 fewer cases; 95% CI 0.37–1.19; *P* < 0.001); standard of care issues (1.37 fewer cases; 95% CI 0.49–2.26; *P* = 0.002); sexual misconduct (1.65 fewer cases; 95% CI 0.90–2.40; *P* < 0.001); miscellaneous findings (0.80 fewer cases; 95% CI 0.11–1.50; *P* = 0.020); mental illness (0.06 fewer cases; 95% CI 0.01–0.12; *P* = 0.025); inappropriate prescribing (0.74 fewer cases; 95% CI 0.15–1.33; *P* = 0.010); and conviction of a crime (0.33 fewer cases; 95% CI 0.00–0.65; *P* = 0.048) (Table 3, Figure 2). No significant rate differences were found with respect to unclear violations, fraudulent behavior/prevarication, or offenses involving drugs and/or alcohol (all rate differences smaller than 0.32 events per 10,000 physician years) (Table 3, Figure 2).

**TABLE 3:**
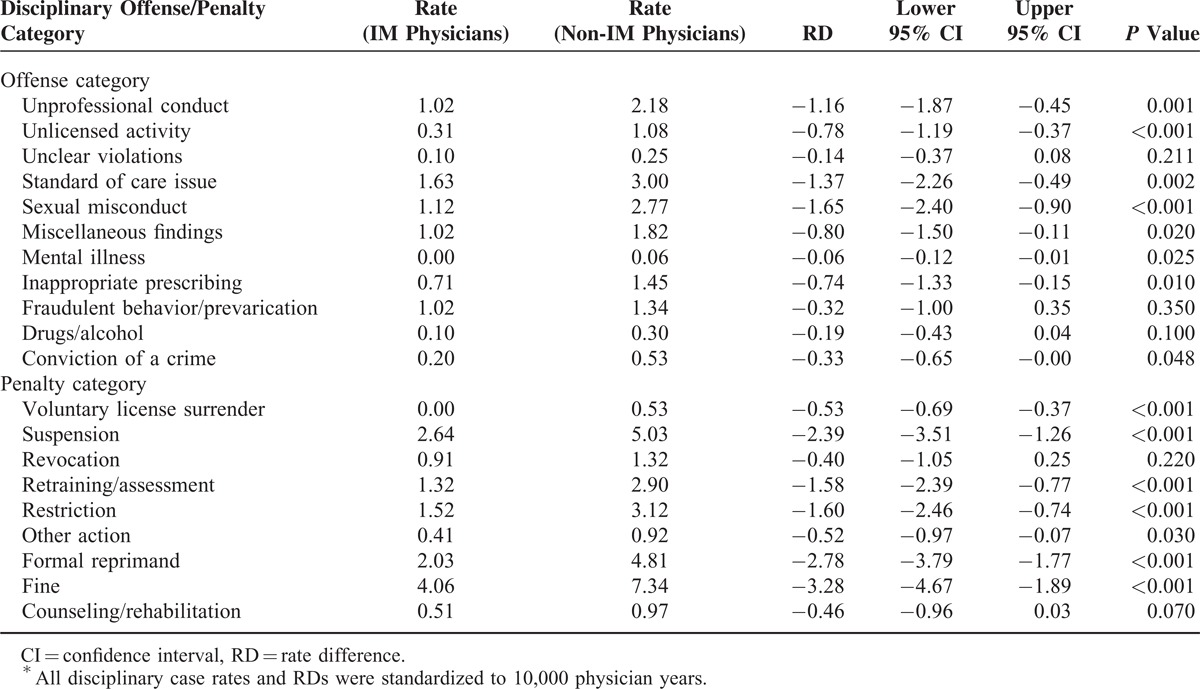
Disciplinary Rates^∗^ and Rate Differences (RDs) for Internal Medicine (IM) Versus Non-IM Physicians, by Specific Offense and Penalty Type (2000–2013)

**FIGURE 3:**
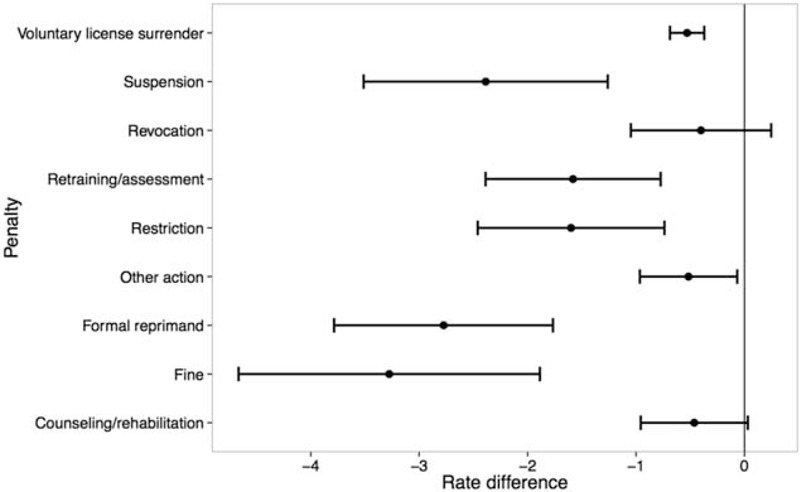
Rate differences (RDs) by penalty type for internal medicine (IM) physicians, as compared with non-IM physicians^∗^ (2000–2013). ^∗^Error bars denote 95% confidence intervals.

With respect to specific penalty types, IM physicians were significantly less likely than other physicians to be handed the following penalties: voluntary license surrender (0.53 fewer cases; 95% CI 0.37–0.69; *P* < 0.001); suspension (2.39 fewer cases; 95% CI 1.26–3.51; *P* < 0.001); retraining/assessment (1.58 fewer cases; 95% CI 0.77–2.39; *P* < 0.001]; restriction (1.60 fewer cases; 95% CI 0.74–2.46; *P* < 0.001]; other actions (0.52 fewer cases; 95% CI 0.07–0.97; *P* = 0.030); formal reprimand (2.78 fewer cases; 95% CI 1.77–3.79; *P* < 0.001; or fine (3.28 fewer cases; 95% CI 1.89–4.67; *P* < 0.001) (Table 3, Figure 3). No significant differences were found with respect to revocation or mandated counseling/rehabilitation (all rate differences smaller than 0.46) (Table 3, Figure 3).

## DISCUSSION

Our study of all physicians disciplined in Canada from 2000 to 2013 found that IM physicians underwent disciplinary proceedings at a significantly lower rate than non-IM physicians. Disciplinary rates were low for all physicians (9.6 cases per 10,000 physician years); however, as a group, IM physicians had disciplinary rates of approximately half that of other physicians. In addition, when specific disciplinary offense and resultant penalties were considered, IM physicians were significantly less likely to be disciplined for most categories of discipline, including unprofessional conduct, unlicensed activities, standard of care issues, sexual misconduct, miscellaneous findings, mental illness-related offenses, inappropriate prescribing and conviction of a criminal offense. With respect to specific penalties, IM physicians were less likely to undergo voluntary license surrender, suspension, mandated retraining/assessment, license restriction, other penalties, formal reprimand, or fine.

One of the strengths of our study is that, to our knowledge, this is the first study to investigate disciplinary cases by IM physicians, as most previous literature focused on baseline demographics of all physicians or has focused specifically on subspecialties such as anesthesia or psychiatry.^1–7^ Second, our study period of 14 years was sufficiently large to capture what are relatively infrequent events of misconduct. Third, our results are the first to subcategorize disciplinary action against IM physicians into subspecialty, and therefore contribute to the limited existing data on disciplined IM physicians.

Our finding that IM physicians, on average, represent 10.8% of all disciplined physicians in the country is in keeping with previous studies that have shown that this population comprises 1.3–15% of all disciplined physicians.^1–4^ Potential explanations for this moderate variability may relate to the scope and nature of the practice of internal medicine across jurisdictions. IM physicians are less likely to be disciplined compared with psychiatrists, obstetricians and gynecologists, and family physicians, even after adjusting for age, gender, board certification, and international medical training.^1^ Data from Canada, Australia, and New Zealand have also shown similar results.^3–7^ However, most previous research has focused on baseline demographics of disciplined physicians, or other specialties.^1–4,6,7,9^

In our dataset of disciplined IM physicians, almost all were male. That nearly three-quarters of Canadian IM physicians are male may partially explain this effect.^12^ However, previous literature has shown that male physicians have higher disciplinary rates—in 1 study, up to 4 times more than their female counterparts.^1–7^ Some argue that it may not be whether a physician is male or female, but another variable, such as poor communication skills, that drives patient complaints and subsequent disciplinary action.^14–18^ Similarly, others have also described a link between malpractice suits, misconduct, and poor communication skills.^19–21^

Patient-specific factors may also contribute to lower rates of reported disciplinary action. In Canada, IM and its subspecialties are generally consultative in nature. Compared with primary care, IM patient encounters may differ in the frequency and intensity of care, which may account for lower disciplinary rates compared to the U.S. In these situations, there may be fewer opportunities for certain types of misconduct to occur, particularly those involving boundary issues, sexual misconduct, and inappropriate relationships—which may in part, explain our finding that IM physicians and lower rates of disciplinary action for sexual offenses.^6^

Physician-specific factors such as work environment may also contribute to our findings. The majority of misconduct typically occurs in ambulatory over inpatient settings.^4^ Indeed, the most frequently disciplined specialties in Canada were family medicine and psychiatry—both ambulatory-based specialties in which longstanding physician–patient relationships are more common.^5–7^ Potential mechanisms for increased episodes of misconduct in an ambulatory care setting include increased prolonged psychosocial interaction.^6^ Moreover, the complex nature and acuity of illness of hospitalized IM patients often requires multispecialty coordinated care between several physicians and members of the allied health care team. It may be comparatively difficult in a hospitalized IM patient to ascertain which health care provider is making primary decisions regarding care, and therefore, more challenging to identify certain types of misconduct (eg, failure to maintain standards of care or inappropriate prescribing).

We acknowledge several limitations to our work. Published accounts of disciplinary action are an imperfect surrogate that may underestimate true incidence rates of misconduct. Our database does not capture unreported incidences of misconduct, nor does it include misconduct instances that are settled outside the public domain (ie, in civil legal proceedings). Physicians who voluntarily relinquish their practice license before any accusation or suspicion of physician misconduct are also not included. Disciplinary proceedings may be driven by multiple factors that may be, in part, independent of misconduct, such as patient dissatisfaction, and thus may not be a perfect surrogate for physician misconduct. However, at this time, this is the most complete method of accounting for this population of physicians. Second, while our database is as inclusive as possible, there were instances where information was not available. Certain disciplinary cases maintained physician anonymity; in these instances, key demographic information was missing regarding identity, gender, year of graduation, and in most cases, specialty. However, information regarding type of disciplinary offense and penalties were still made available, and overall, this represented a small number of our population (24 or 3.1% of all disciplined physicians).

Second, we were unable to account for any disciplinary cases in 3 of Canada's jurisdictions: Nunavut, Yukon, and Northwest Territories, as well as in certain provinces before online publication of that province's discipline summaries, namely New Brunswick, Prince Edward Island and Newfoundland and Labrador before 2007, as well as Alberta before 2002. Thus, overall, we may have underestimated the true population of all disciplined physicians.

Finally, our findings describe the national experience of Canada. While they may not be directly reflective of the experiences in other countries, we would argue that there are more similarities in the discipline system by professional colleges between countries than in the state and federal criminal legal systems.^22,23^

Our results showed that internal medicine physicians have lower rates of documented disciplinary action than other physicians. Although the absolute number of disciplinary proceedings is low, any instance of physician misconduct warrants concern. It remains to be seen whether there is a population of at-risk clinicians who may be detected early, as well as whether there are any interventions that may obviate misconduct in the first place. We believe that to improve patient quality of care and safety, it should be a priority for the medical profession to examine these issues further to eliminate as many instances of physician misconduct as possible. In this way, prevention may be the best medicine.

## References

[1] Kohatsu ND Gould D Ross LK Characteristics associated with physician discipline: a case control study. *Arch Intern Med* 2004; 164:653–658. 15037494 10.1001/archinte.164.6.653

[2] Khaliq AA Dimassi H Huang CY Disciplinary actions against physicians: who is more likely to get disciplined? *Am J Med* 2005; 118:773–777. 15989912 10.1016/j.amjmed.2005.01.051

[3] Morrison J Wickersham P Physicians disciplined by State Medical Board. *JAMA* 1998; 279:1889–1894. 9634260 10.1001/jama.279.23.1889

[4] Elkin KJ Spittal MJ Elkin DJ Doctors disciplined for professional misconduct in Australia and New Zealand, 2000–2009. *Med J Austral* 2009; 194:452–456. 21534900 10.5694/j.1326-5377.2011.tb03058.x

[5] Alam A Klemensberg J Griesman J The characteristics of physicians disciplined by professional colleges in Canada. *Open Med* 2011; 5:166–172. PMC3345379 22567070

[6] Alam A Kuryak P Klemensberg J The characteristics of psychiatrists disciplined by professional colleges in Canada. *PLoS One* 2012; 7:1–5. 10.1371/journal.pone.0050558 PMC3509088 23209779

[7] Alam AQ Khan J Liu J Characteristics and disciplinary rates of anesthesiologists disciplined by professional colleges in Canada. *Can J Anesth* 2013; 60:1013–1019. 23897490 10.1007/s12630-013-0006-8

[8] Donaldson LJ Doctors with problems in an NHS workforce. *BMJ* 1994; 308:1277–1282. 8205022 10.1136/bmj.308.6939.1277 PMC2540199

[9] Reich JH Maldonado J Empirical findings on legal difficulties among practicing psychiatrists. *Annals Clin Psyc* 2011; 23:297–307. 22073387

[10] http://www.acponline.org/patients_families/about_internal_medicine/ Accessed September 2014.

[11] CAPER (Canadian Post-M.D. Education Registry). Annual census of post-M.D. trainees 2009–2012. Ottawa: Association of Faculties of Medicine of Canada; 2010 Accessed September 2014: www.caper.ca/download_publications_en.php

[12] Canadian Institute for Health Information. Supply, distribution and migration of Canadian physicians [2000–2012.]. Accessed September 2014: https://secure.cihi.ca/estore/productSeries.htm?pc = PCC34

[13] Rajan C *The Canadian Medical Directory. Scott's Directories*. Volumes 1954–2013.

[14] Firth-Cozens J Doctors with difficulties: why so few women? *Postgrad Med J* 2008; 84:318–320. 18644923 10.1136/pgmj.2008.068478

[15] Montini T Noble AA Stelfox HT Content analysis of patient complaints. *Int J Qual Health Care* 2008; 20:412–420. 18801751 10.1093/intqhc/mzn041

[16] Van Mook WNK Gorter SL Kieboom W Poor professionalism identified through investigation of unsolicited healthcare complaints. *Postgrad Med J* 2012; 88:443–450. 22595102 10.1136/postgradmedj-2011-130083

[17] Anderson K Allan D Finucane P A 30-month study of patient complaints at a major Australian hospital. *J Qual Clin Practice* 2001; 21:109–111. 10.1046/j.1440-1762.2001.00422.x 11856405

[18] Roter D Lipkin M Korsgaard A Sex differences in patients’ and physicians’ communication during primary care medical visits. *Med Care* 1991; 29:1083–1093. 1943269 10.1097/00005650-199111000-00002

[19] Levinson W Roter D Mullooly J Physician-patient communication: the relationship with malpractice claims among primary care physicians and surgeons. *JAMA* 1997; 277:533–559. 10.1001/jama.277.7.553 9032162

[20] Hickson GB Federspiel CF Pichert JW Patient complaints and Malpractice Risk. *JAMA* 2002; 287:2951–2957. 12052124 10.1001/jama.287.22.2951

[21] Stelfox HT Gandhi TK Orav EJ The relation of patient satisfaction with complaints against physicians and malpractice lawsuits. *Am J Med* 2005; 118:1126–1133. 16194644 10.1016/j.amjmed.2005.01.060

[22] Borow M Levi B Glekin M Regulatory tasks of national medical associations – international comparison and the Israeli case. *Israel J Health Pol Res* 2013; 2:8. 10.1186/2045-4015-2-8 PMC3598843 23425333

[23] Wallace E Lowry J Smith SM The epidemiology of malpractice claims in primary care: a systematic review. *BMJ Open* 2013; 3:e002929. 10.1136/bmjopen-2013-002929 PMC3693415 23869100

